# Determination of Lincomycin in Milk Using Cu-Based Metal-Organic Framework Adsorbent and Liquid Chromatography-Tandem Mass Spectrometry

**DOI:** 10.3390/molecules28145307

**Published:** 2023-07-10

**Authors:** Hanle Li, Jinhai Wu, Jialei Bai, Jianhu Wu, Jin Wu

**Affiliations:** 1 College of Food Science Sciences, Shanxi Normal University, Taiyuan 030000, China; lhl961029@outlook.com (H.L.);; 2 Tianjin Key Laboratory of Risk Assessment and Control Technology for Environment and Food Safety, Tianjin Institute of Environmental and Operational Medicine, Tianjin 300050, China

**Keywords:** Cu-based metal-organic framework, liquid chromatography-tandem mass spectrometry, sampling preparation technique, solid-phase extraction, lincomycin

## Abstract

Antibiotic drug residues can adversely affect the human body. Lincomycin is a common veterinary drug that can form residues in foods of animal origin. However, the detection of trace residue levels of lincomycin residues in real samples is challenging. Here, a simple solid phase extraction (SPE) method was developed for the enrichment of lincomycin from cow milk samples before its detection by high-performance liquid chromatography-tandem mass spectrometry (HPLC-MS/MS). The adsorbent used in the SPE was a Cu-based metal-organic framework (Cu-MOF) prepared by the solvothermal synthesis approach. The prepared MOFs were characterized using scanning electron microscopy (SEM), Fourier-transform infrared spectroscopy (FT-IR), X-ray diffractometry (XRD), differential thermogravimetric analysis (TG-DTA), and N2 adsorption-desorption experiments. The adsorption capacity (adsorption equilibrium, extraction time, pH), and elution solvent parameters were investigated. Under the optimized conditions of the HPLC-MS/MS method, lincomycin was detected in the linear range of 10–200 g/L with a detection limit of 0.013 ng/mL. Commercial milk samples were spiked with lincomycin, and a recovery rate between 92.3% and 97.2% was achieved. Therefore, the current method can be successfully applied for the enrichment and determination of lincomycin from milk samples.

## 1. Introduction

Antibiotics differ from common drugs in the manner of their poisoning and death. Very small doses of antibiotics may cause poisoning and death because of the manner of administration and the contraindications of drug formulations [1]. Lincomycin is a lincosamide antibiotic produced by Streptomyces lincomycetes. This antibiotic binds to the 50S ribosomal subunit on the ribosome and blocks prokaryotic translation, thereby inhibiting protein synthesis in the bacterial cells [2]. Lincomycin is a common antibiotic used worldwide. It is generally used as a veterinary drug and remains in animal-derived foods, causing allergies, toxicity, and other adverse symptoms. Therefore, the use of lincomycin has been restricted in various countries and regions. The European Union (EU) has established the limits for lincomycin content in foodstuffs, wherein the maximum residue levels (MRL) for all producing species are 100 g/kg (muscle), 50 g/kg (fat), 500 g/kg (liver), 1500 g/kg (kidney), 150 g/kg (milk), and 50 g/kg (eggs) [3]. It is essential to develop a simple, rapid, and sensitive extraction and detection method for lincomycin [4,5].

Food sample substrates are complex and involve complicated pretreatment steps before detection. Hence, extraction and separation steps are necessary. The extraction and separation techniques commonly used in traditional sample pretreatment include Soxhlet extraction, supercritical fluid extraction [6], sonication-assisted extraction, etc. [7]. However, these methods require a large number of samples, consume large amounts of organic solvents, and are complicated to operate, thus generating a large number of toxic waste solvents [8,9]. Hence, sample preparation techniques are the focus of recent research. Solid-phase extraction (SPE) is a simple sample preparation method with the advantages of high extraction recovery, high enrichment rate, and easy operation [10]. Particularly, adsorbents and other related materials can be recycled, greatly reducing the cost of disposal of waste pollutants [4,8,11]. Traditional adsorbents, such as activated carbon, chitosan, natural zeolite, and silicon microspheres, are commonly used by the wastewater industry for water treatment. These materials possess poor adsorption capacity and, hence, are generally suitable for low concentrations of dye or heavy metal contamination. The MOF (metal-organic framework) coordination polymer has developed rapidly in the past decade. It is advantageous in terms of high porosity, low density, a large specific surface area, regular and adjustable pore sizes, and a variety of topological structures [12]. It is widely used in photoelectric catalysis, gas separation, energy storage sensing, and the adsorption of organic pollutants [2,13,14]. An important advantage of MOF, in contrast to traditional adsorbents, is the ability to regulate their properties through the selection of ligands and metal ions of different structures and various methods of combining them to obtain different diameter holes and change the size of the cavity in the three-dimensional structure, which greatly improves the adsorption efficiency toward target pollutants [15].

Furthermore, the ease of synthesis of MOF makes it unique in the selection of adsorbents. Therefore, as a new adsorbent, MOF has significant advantages and broad application prospects in the solid-phase extraction of antibiotic pollutants [16,17].

Cu-based metal-organic frameworks (Cu-MOFs) are composed of oxygen, nitrogen, other organic ligands, and copper ions that are self-assembled by coordination polymers. In the structures of Cu-MOFs, the arrangement of organic ligands and metal ions shows obvious directions, which can form different pore structures and have different adsorption, optical, and electromagnetic properties. Therefore, Cu-MOFs demonstrate great potential and attractive prospects in the field of modern materials science [18].

Presently, lincomycin is chiefly detected using high-performance liquid chromatography (HPLC) [19,20], gas chromatography-mass spectrometry (GC-MS) [4,21,22] and liquid chromatography-tandem mass spectrometry (HPLC-MS/MS) [23,24]. HPLC-MS/MS overcomes the background interference, and its multi-reaction detection mode (MRM) improves the signal-to-noise ratio. Thus, high sensitivity can be achieved for complex samples. Currently, this is the ideal method for drug residue analysis.

In this study, Cu-MOFs were prepared using solvothermal methods, and the products were characterized. Cu-MOF functioned as an effective adsorbent to extract lincomycin from milk, and the important factors that affected the adsorption efficiency were systematically optimized. Finally, an HPLC-MS/MS method was established for lincomycin detection. Additionally, the SPE-HPLC-MS/MS method was validated under the optimized conditions and applied for analyzing lincomycin in different milk samples.

## 2. Results and Discussion

### 2.1. Characterization of Cu-MOFs

**Figure 1:**
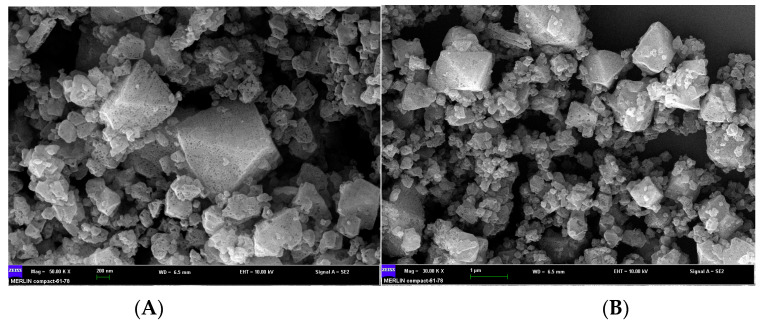
(**A**) high-magnification (50K ×) SEM. image of the Cu-MOFs; (**B**) low-magnification (30K ×) SEM. image of Cu-MOFs.

**Figure 2:**
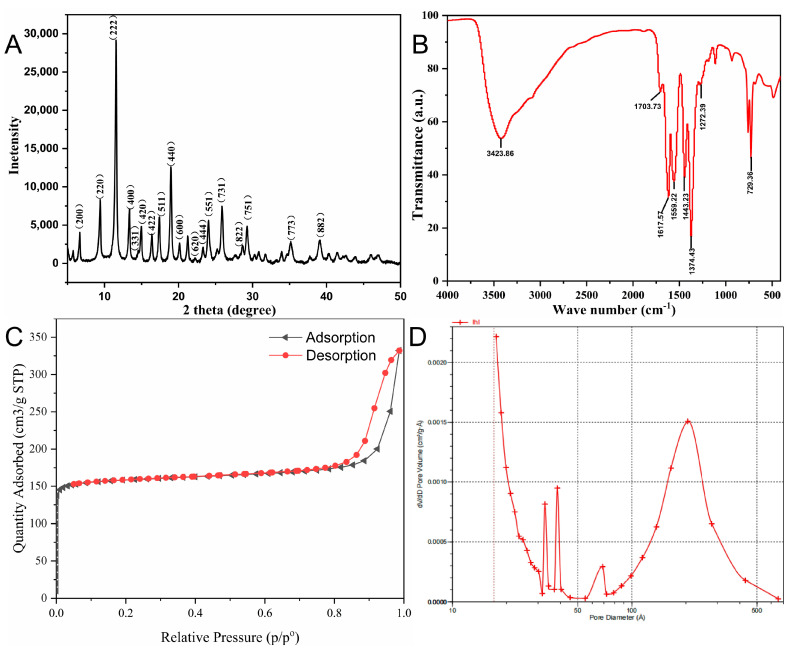
(**A**) XRD patterns of Cu–MOFs, (**B**) FT-IR spectra of Cu–MOFs, (**C**) N_2_ adsorption-desorption isotherms of Cu–MOFs, (**D**) Pore size distribution of Cu–MOFs.

The morphology of Cu-MOFs was characterized by scanning electron microscopy (SEM). The Cu-MOF exhibited a typical octahedral shape with a pore structure on the surface (Figure 1) [25,26]. The XRD pattern of the as-prepared Cu-MOFs layer is shown in Figure 2A. As-prepared Cu-MOFs consist of a face-centered cubic (FCC) crystal lattice, with reflections of (222), (400), (331), (420), (422), (511), (440), (600), (444), (551), (731), (822), (751), (773), and (882) planes at 2 of 11.56°, 13.38°, 14.54°, 14.96°, 16.38°, 17.42°, 18.96°, 20.14°, 23.32°, 24.06°, 25.86°, 28.62°, 29.26°, 35.16°, and 39.1°; these results were consistent with the literature reports [27,28,29]. Thus, the results implied the successful synthesis of Cu-MOFs crystals with a topological structure.

The functional group composition of the prepared Cu-MOFs was characterized by Fourier transform infrared spectroscopy (FTIR). As revealed in Figure 2B, the sample exhibit the characteristic asymmetric stretching vibrations of carboxylate groups in the range of 1559–1443 cm^−1^ and the symmetric stretching vibrations around 1374 cm^−1^. However, in the same wave number range, the vibration band could also result from the stretching vibrations of the benzene ring as well as the deformation vibration of water molecules. Beside bands related to COO- groups, the band at 1443 cm^−1^ is ascribed to the C-C vibration in the aromatic ring. A wide peak at 3423 cm^−1^ was due to the OH groups stretching vibration of water molecules. The presence of characteristic absorption bands of free BTC at 1703–1617 cm^−1^ and at 1272 cm^−1^, is due to the stretching vibration of the C=O group and the C-OH group, respectively. As well, the peak around 730 cm^−1^ is related to the bending vibration of C-H. These results were consistent with the reported results [25,30], confirming the successful synthesis of the Cu-MOFs materials.

To study the specific surface area and pore size of the Cu-MOF samples, N_2_ adsorption-desorption isotherm curves were tested, and they displayed a type IV isotherm (Figure 2C). The adsorption capacity increases rapidly at a lower relative pressure, and the saturation value appears when the relative pressure reaches a certain level. At an approximate relative pressure (P/P_0_) of 0.8, capillary condensation occurred in the pores of the material, and hysteric loops appeared in the adsorption-desorption isotherm curve. It is proven that the material has microporous and mesoporous structures. As well, the specific surface area (630.08 m^2^/g) and pore volume (0.30 cm^3^/g) of Cu-MOFs porous materials were obtained. The pore size distribution is shown in Figure 2D, and the average pore size within the mesoporous range is about 20 nm according to the BJH calculation. Additionally, the large specific surface area and pore volume of the Cu-MOFs would provide more space and sites for target molecules.

**Figure 3:**
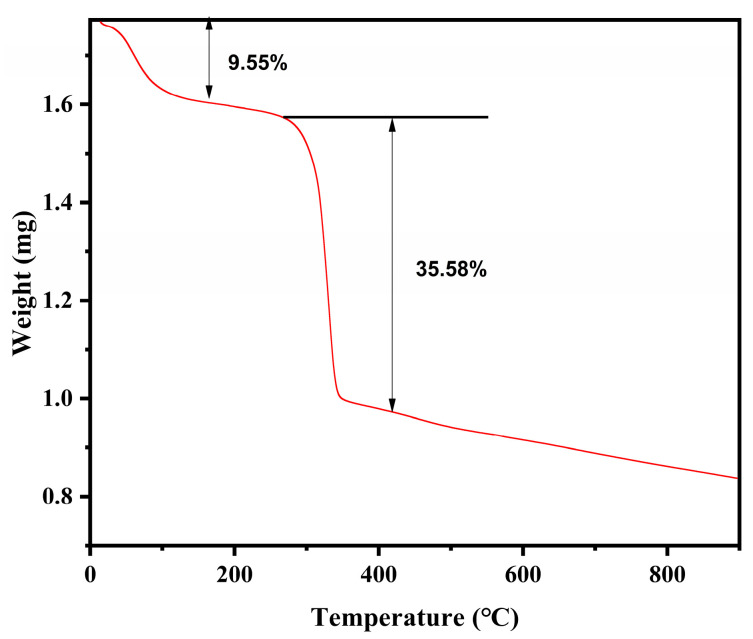
Comprehensive Thermal Analysis of Cu-MOFs.

Further, the thermal stability of Cu-MOFs was studied by the TG-DSC method. Under the nitrogen atmosphere, the heating rate was 5 °C/min, and the quality was monitored from room temperature to 900 °C. The weight of the Cu-MOFs decreased slightly with an increase in the temperature around 100 °C (Figure 3). This could be due to the evaporation of water molecules in the material during the heating process. When the temperature was increased from 100 °C to 300 °C, there was no change in the weight of the Cu-MOFs. Further increase in the temperature destroyed the Cu-MOF structure resulting in a 35.58% decrease in weight. Therefore, Cu-MOFs show superior thermodynamic stability in the range of 300 °C.

### 2.2. Adsorption Experiment of Cu-MOFs

#### 2.2.1. Saturated Adsorption Capacity

**Figure 4:**
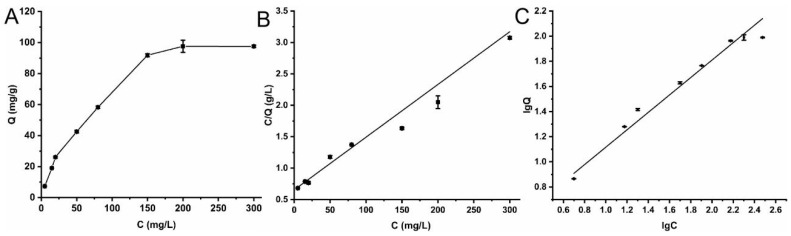
Adsorption equilibrium of lincomycin by Cu-MOFs, (**A**) Adsorption equilibrium curve, (**B**) Langmuir isothermal adsorption fitting curves, (**C**) Freundlich isothermal adsorption fitting curves.

The adsorption equilibrium curve of Cu-MOFs for lincomycin is shown in Figure 4A. At low lincomycin concentrations, the adsorption capacity increased rapidly with increasing concentration. When the lincomycin concentration was increased to 200 mg/L, the adsorption amount of lincomycin no longer changed. Further increases in the concentrations did not significantly increase the adsorption amount, indicating that the reaction had reached equilibrium. The adsorption of antibiotics at different concentrations was investigated according to the Langmuir (Figure 4B) and Freundlich (Figure 4C) models [31,32].

(1) $$\frac{C_{e}}{Q_{e}}=\frac{1}{Q_{\max}}C_{e}+\frac{1}{Q_{\max}\times b}$$

(2) $$\mathrm{lg}Q=\frac{1}{n}\mathrm{lg}C+\mathrm{lg}K_{f}$$

where *C_e_* (mg/L) is the concentration of the adsorbate at adsorption equilibrium. *Q_e_* (mg/g) is the amount of adsorbed pharmaceuticals on the adsorbent at equilibrium (mg/g), *Q*_max_ (mg/g) is the saturated adsorption capacity of the adsorbent at adsorption equilibrium, *b* is the Langmuir constant, and *n* and *K_f_* are the Freundlich constants.

As seen in Table 1, the R^2^ (=0.98) value of the Langmuir model was higher, indicating a monolayer adsorption process. The adsorption capacity of all sites on the solid surface was identical. When the concentration of the target substance was low, the antibiotic quickly adsorbed on the surface of the material and occupied the surface adsorption site. Upon increasing the target concentration, the surface adsorption site gradually became saturated and lost its adsorption effect. According to the Langmuir isothermal adsorption equation, the saturated adsorption capacity of lincomycin was calculated to be about 131.41 mg/g. The adsorption capacity is higher than the adsorption capacity of chitosan and magnetic nanomaterials for antibiotics reported in the literature [33,34].

#### 2.2.2. Adsorption Kinetics

**Figure 5:**
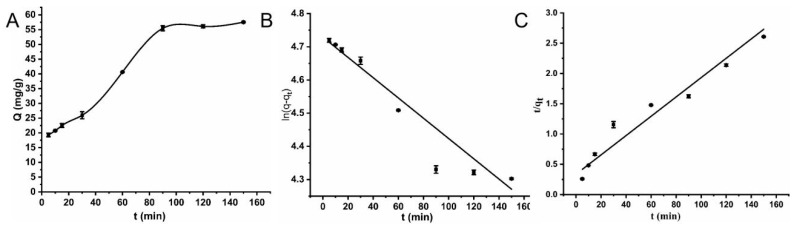
The adsorption kinetic results of lincomycin by the Cu-MOFs, (**A**) The adsorption kinetic curve, (**B**) The pseudo-first-order kinetic model, (**C**) The pseudo second-order kinetic model.

The effect of different adsorption times on lincomycin adsorption capacity was studied with 2.0 mg Cu-MOFs (Figure 5A). When the oscillation time was between 5 and 90 min, the adsorption capacity increased with an increase in the oscillation time. The adsorption took nearly 90 min to attain equilibrium but continued for 150 min, to check the fluctuations in adsorption equilibrium, but the adsorption stayed constant. To further analyze and calculate the kinetic parameters, the experimental data were fitted by two kinetic models: the pseudo-first-order (3) and pseudo-second-order (4) kinetic models [31,35]:

(3) $$\ln(q-q_{t})=\ln q-k_{1}t$$

(4) $$\frac{t}{q_{t}}=\frac{1}{k_{2}\times q^{2}}+\frac{t}{q}$$

where *q_t_* (mg·g^−1^) and *q* (mg·g^−1^) are the amounts of lincomycin adsorbed at time *t*(min) and the amount at adsorption equilibrium, respectively; and *k*_1_ (min^−1^) and *k*_2_ (g·mg^−1^·min^−1^) are the pseudo-first-order and pseudo-second-order rate constants, respectively.

Although adsorption was very quick and spontaneous at the start, it progressively slowed down until equilibrium was established. Although adsorption was very quick and spontaneous at the start, it progressively slowed down until equilibrium was established. In the case of Cu-MOFs, there was a big difference in the values of both *k*_1_ and *k*_2_ but the experimental values were very close to the pseudo-second-order kinetics model such as *q*_1_ was 112.97 mg·g^−1^ and *q*_2_ was 68.21 mg·g^−1^ compared to experimental values. Consequently, the pseudo-second-order model tailored this process in terms of the association factor (R^2^) being superior to the pseudo-first-order. The adsorption graph for both orders is presented in Figure 5B,C. The data obtained from the experiment and pseudo-first-order and pseudo-second-order kinetics models are enclosed in Table 2. Therefore, it shows that the target reaching the surface of the adsorbent from the solution was controlled by the chemisorption mechanism, and there were two types of binding sites on the surface of the adsorbent [36,37,38,39].

#### 2.2.3. Optimization of the Eluent Solution

**Figure 6:**
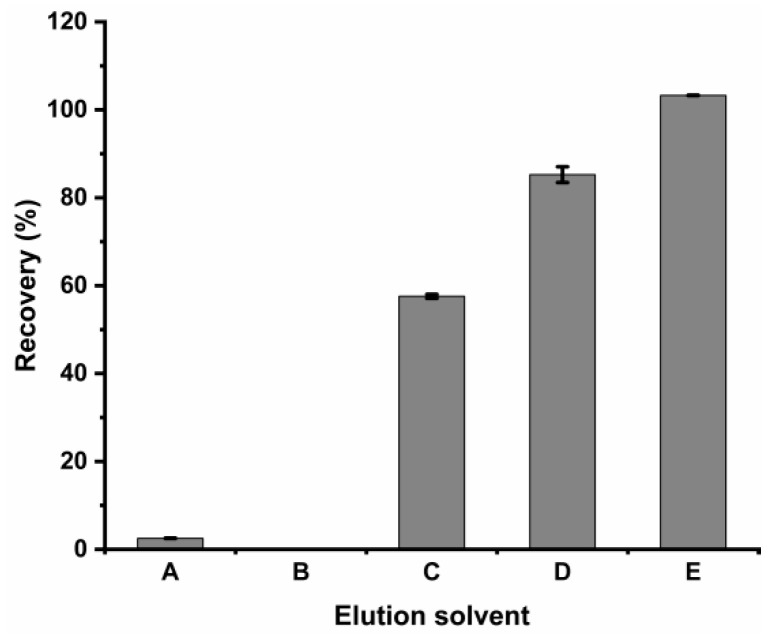
Effect of the solvent type on the recovery of lincomycin (A: MeOH. B: Acetonitrile. C: Water. D: MeOH containing 1% formic acid. E: Acetonitrile containing 1%formic acid).

In this experiment, five elution solvents were investigated (Figure 6). When acetonitrile with 1% formic acid was used as the elution solvent, the recovery rate was almost 100%. The dependence of the adsorption between antibiotics and adsorbents on a variety of interactions could be the probable reason. The hydrogen bonding, electrostatic interactions, and π-π interactions between the antibiotic and the adsorbent were destroyed by adding a small amount of formic acid, and a satisfactory elution effect was achieved. However, when the 1% formic acid in water was used as the eluent, the Cu-MOF material was completely dissolved, and the structure of the material was destroyed. Therefore, 3 mL of acetonitrile with 1% formic acid was selected as the best elution solvent.

#### 2.2.4. Effect of pH on the Adsorption Process

**Figure 7:**
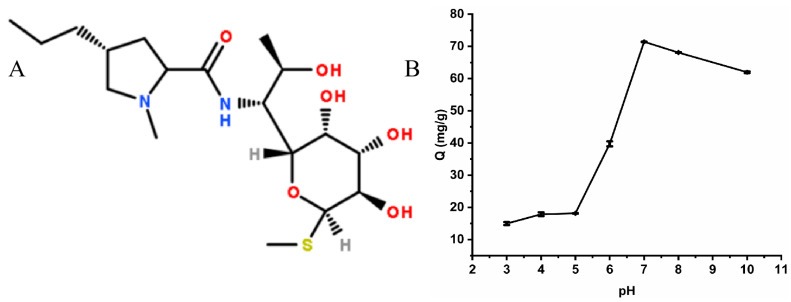
(**A**) Structure of lincomycin, (**B**) pH effects during the adsorption process.

The pH value affects the adsorption process. The charge state on the surface of the material and the chemical form of the antibiotic are both impacted by the pH value of the solution, thus affecting the adsorption performance of the material on the antibiotic. In this study, the adsorption experiments between Cu-MOFs and lincomycin were performed under different pH conditions (pH = 4~10) (Figure 7B). Upon increasing the pH of the solution, the adsorption of Cu-MOFs on lincomycin initially increased and then decreased. The maximum adsorption was achieved at pH 7. The structural formula of lincomycin is shown in Figure 7A. The molecule is unstable under acidic conditions, and the functional groups are easily protonated with a positive charge. Moreover, the functional groups on the surface of the adsorbent are easily protonated under acidic conditions. Therefore, the interaction between lincomycin and the adsorbent was reduced in an acidic solution due to a similar surface charge. As the pH of the solution increased, the functional groups on the adsorbent surface were deprotonated. This increased the negative charge density on the adsorbent surface and enhanced the binding of lincomycin on the adsorbent surface. Additionally, at higher pH values, the lincomycin adsorption capacity was higher due to a decrease in the H-adsorbed competitors on the active site of the adsorbent. The results showed that Cu-MOF adsorbents had excellent adsorption capacity toward lincomycin. This was probably due to the strong hydrogen bond interaction between the adsorbent surface’s COOH functional group and the OH of the lincomycin molecule at pH 7.

### 2.3. Method Validation

#### 2.3.1. Linear Range and Detection Limit

Under optimal experimental conditions, the analytical performance of the developed method was evaluated by establishing calibration curves and calculating the limit of detection (LOD), linear range, relative standard deviation (RSD), and recovery rate. Good linear relationships were obtained in the concentration range of 10~200 μg/L, with correlation coefficients (R) of lincomycin in milk samples exceeding 0.99. The limits of detection (LODs) of lincomycin were determined by considering the signal-to-noise ratio (S/N) = 3.

#### 2.3.2. Detection of Real Samples

To verify the accuracy and precision of the method, three blank milk samples were spiked with 100 μg/L, 500 μg/L, and 1000 μg/L lincomycin, respectively. A recovery of 92.3~97.2% of lincomycin was achieved by this method and the precision (*n* = 3) was 0.25~1.96% (Table 3). Thus, the current method demonstrated good accuracy and precision and could meet the requirements of analysis.

#### 2.3.3. Comparison of the Developed Method with Reported Methods

The developed method was compared with previous reports describing the determination of lincomycin in different real samples (Table 4). From an analytical aspect, the linear range and LOD of the proposed method were approximately similar to or better than those of the reported methods. The added level of lincomycin in this experiment was lower than the maximum residue level in the European Union limit, the method proposed in this study led to greater accuracy and precision compared with other methods (the recoveries of lincomycin were 92.3–97.2%, the RSDs were 0.25–1.96%). Thus, this method represents a promising future for the application of Cu-MOFs in determining lincomycin from complicated cow milk samples. The proposed method requires no expensive equipment, and the materials and reagents are easily available. Some of the previously reported methods require multiple steps and are time-consuming, while the present method is one-step.

## 3. Experimental

### 3.1. Chemicals and Instruments

Cu(NO_3_)_2_·3H_2_O and formic acid were purchased from Merck (Shanghai, China); 1,3,5-benzenetricarboxylic acid (H_3_BTC, 98%) was obtained from Aladdin Reagent Corporation (Shanghai, China). Ultrapure water was purchased from Hangzhou Wahaha Group Co., Ltd. (Hangzhou, China). HPLC-grade acetonitrile and methanol were supplied by Thermo Fisher Scientific (Waltham, MA, USA). Ethyl alcohol was obtained from J&K Chemicals (Beijing, China); Lincomycin (98.8%) powder was obtained from Stanford Chemicals Company (Beijing, China); three milk samples were purchased from the local supermarket and stored under refrigeration (−4 °C) until further use.

The functional groups in the Cu-MOF were investigated using Fourier transform infrared spectroscopy (Nicolet FT-IR 6700, Shanghai Larui Company, Shanghai, China). The morphology of the prepared Cu-MOFs was examined using Scanning electron microscopy (SEM) (VISUCAM PRONM microscope from ZEISS, Jena, Germany). The phase and structure of the prepared Cu-MOFs were characterized using X-ray powder diffraction (XRD, Rigaku, Tokyo, Japan) using SmartLab (9 kW) at angles ranging from 5° to 80°. The heat stability of the Cu-MOFs was evaluated using the Simultaneous thermal analyzer NETZSCH STA 449 F3 Jupiter (Kassel, Germany). The Brunauere–Emmette–Teller (BET) surface area was calculated from the N_2_ adsorption-desorption isotherms at 77 K using an ASAP 2020 Surface Area Analyzer (Mike, GA, USA). The chromatography analysis process was performed with a Waters Company system equipped with Xevo TQ-S micro.

### 3.2. Instrument Conditions

HPLC conditions: ACQUITY UPLC BEH C18 column (50 mm × 2.1 mm, 1.7 μm Waters Company, Milford, MA, USA), Mobile phase A: methanol, mobile phase B: 0.1% formic acid aqueous solution. A previously described elution scheme was adapted, and the elution method was optimized according to the results of the total ion diagram. The elution method obtained is shown in Table 5. The total analysis time was 5.0 min. The flow rate was 0.3 mL/min, and the injection volume was 2 μL.

Mass spectrometric conditions: ionization mode: ESI+, Ionization voltage: 3.0 kV, taper hole voltage: 32 V; Ion source temperature: 150 °C, desolventing temperature: 350 °C, desolventing gas velocity: 650 L/h, impact chamber pressure: 3.6 × 10^−5^ Pa; the MRM analysis.

### 3.3. Synthesis of Cu-MOFs

Cu-MOFs were prepared by the solvothermal method according to the previous literature with modifications [21,22]. Cu (NO_3_)_2_·3H_2_O (1.05 g) was added to 30 mL (*v*/*v*, 1:1) water-ethanol mixture, followed by the addition of H_3_BTC (0.5 g). The solution was transferred into the lining of Poly Tetra Fluoro Ethylene (PTFE) and then sealed in the autoclave for reaction at 120 °C for 24 h. After cooling to room temperature, the product was collected and stirred in a water-ethanol mixture (*v*/*v*, 1:1) for 5 min. The supernatant was centrifuged and discarded. Finally, the product was dried in a vacuum drying oven at 80 °C for 6 h.

### 3.4. Adsorption Equilibrium and Adsorption Kinetics Experiments

The standard solutions of lincomycin were prepared at 5, 15, 20, 50, 80, 150, 200, and 300 mg/L. Next, 2.0 mg of Cu-MOFs were dispersed in 3 mL of these lincomycin standard solutions of different concentrations. The two components were allowed to be mixed at 25 °C and 200 r/min for 24 h. Later, the mixture was centrifuged at 6000 r/min for 8 min and the supernatant was collected and filtered through a 0.22 µm microporous filtration membrane. The concentration of lincomycin in the supernatant was determined by HPLC-MS/MS.

In the adsorption kinetics experiments, 2.0 mg of Cu-MOFs were dispersed in a series of 3 mL solutions containing 50 mg/L of the lincomycin standard. Subsequently, the mixture was incubated for 3 h, and the remaining lincomycin concentration was detected at different points in time. The supernatant was collected by centrifugation at 6000 r/min for 8 min. The supernatant was then filtered through a 0.22-m microporous membrane. The concentration of lincomycin in the supernatant was determined by HPLC-MS/MS.

### 3.5. Optimization of the Eluent

The desorption efficiency of lincomycin on Cu-MOFs will depend on the type of elution solvent used. Methanol, acetonitrile, pure water, methanol with 1% formic acid, and acetonitrile containing 1% formic acid were selected as the elution solvents in this experiment. The amount of adsorbent, the adsorption time, and the elution volume were 2 mg, 12 h, and 3 mL, respectively. Five elution solvents were investigated.

### 3.6. Effect of pH on the Adsorption Process

For this experiment, 2.0 mg of Cu-MOFs were dispersed in 3 mL of a solution containing 50 mg/L of the antibiotic solution. NaOH (0.1 mol/L) and HCl (0.1 mol/L) solutions were added to adjust the pH of the solutions (4–10). After oscillating at 25 °C for 12 h, the supernatant was collected by high-speed centrifugation involving speeds above 7000 r/min and the concentration of the antibiotics was determined.

### 3.7. Pretreatment of the Milk Samples and SPE-HPLC-MS/MS Method for Determination of Lincomycin in Cow Milk Samples

The three milk samples (A, B, and C) were obtained from local supermarkets in Tianjin, China. The milk samples were extracted with two volumes of acetonitrile. The mixture was refrigerated for 20 min and then centrifuged at 12,000 r/min for 10 min to remove the precipitate. The supernatant was stored at 4 °C until further analysis.

**Figure 8:**
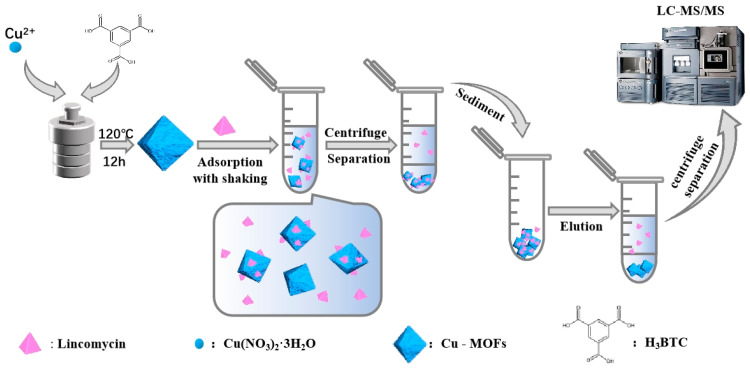
Schematic of the SPE-HPLC-MS/MS procedure for lincomycin extraction from milk samples based on Cu-MOFs.

The schematic of SPE-HPLC-MS/MS and the extraction procedure are described in Figure 8. A fixed volume of the standard analyte solution was added to 10 mL of the milk extract samples, and 2.0 mg of the Cu-MOFs was homogeneously dispersed into the sample solution. The extraction was performed simultaneously with vortex oscillation for 12 h. Subsequently, high-speed spinning centrifugation was applied to separate the adsorbent from the sample solution. The sorbents were then eluted with 3 mL of 1% formic acid in acetonitrile with oscillation for 1.5 h. Finally, the collected eluent was filtered through a 0.22 µm filter membrane for HPLC-MS/MS analysis.

## 4. Conclusions

In this study, Cu-MOFs were prepared by the solvothermal method and successfully applied for the first time for the detection of lincomycin in milk samples. The adsorption capacity of lincomycin was 131.41 mg/g, which followed the Langmuir isothermal adsorption equation and the pseudo-second-order adsorption kinetics model. Cu-MOFs exhibited the best adsorption performance at pH = 7 and exhibited optimal extraction efficiency, enrichment and stability. The method was advantageous in terms of good precision, low detection limit, high recovery rate, and easy operation. Therefore, Cu-MOF is a promising adsorbent material for the enrichment and extraction of other antibiotics from different samples.

## Figures and Tables

**Table 1:** Parameters for Langmuir and Freundlich adsorption isotherm fitting curve.

	Langmuir			Freundlich		
	*b* (L·mg^−1^)	*Q* (mg·g^−1^)	R^2^	*K_f_*/(mg·g^−1^·(L·mg^−1^)^1/*n*^)	*n*	R^2^
Lincomycin	0.012	131.41	0.98	4.256	1.55	0.97

**Table 2:** Fitting parameters of adsorption kinetics.

	The Pseudo First Order			The Pseudo Second Order		
	*k*_1_/min^−1^	*q*/(mg·g^−1^)	R^2^	*k*_2_/(g·mg^−1^·min^−1^)	*q*/(mg·g^−1^)	R^2^
Lincomycin	0.00329	112.974	0.96	0.00051	68.2128	0.99

**Table 3:** Recoveries of spiked milk samples using the current method (*n* = 3).

Simple	Spiked (μg/L)	Recovery/%	RSD/%
Milk A	100	92.3	0.83
	500	96.9	0.25
	1000	97.0	1.81
Milk B	100	92.3	1.16
	500	96.7	1.29
	1000	96.9	0.89
Milk C	100	93.1	1.49
	500	97.1	1.96
	1000	97.2	0.97

**Table 4:** Comparison of the method developed in the present study with other relevant reported methods for the determination of lincomycin.

Method	Sample	Adsorbent	LOD	Recovery (%)	Ref.
LC-MS/MS	eggs	NO	0.5 μg/kg	86.00–111.00	[40]
ASE-SPE-GC–MS/MS	poultry muscles and pork	diatomaceous earth	4.6 μg/kg	79.70–94.20	[4]
PPE-LC-MS/MS	human blood	NO	0.2 ng/mL	72.70–84.13	[2]
FMIA	Milk, Honey, Beef, and Swine Urine	NO	0.69 ng/mL	73.92–120.50	[41]
SPE-HPLC-MS/MS	Milk	Cu-MOFs	0.13 ng/mL	92.30–97.20	This work

**Table 5:** Elution procedure.

Time/min	Mobile Phase A/%	Mobile Phase B/%	Flow Rate/(mL·min^−1^)
Initial	65	35	0.300
1.00	65	35	0.300
1.20	100	0	0.300
1.50	0	100	0.300
2.00	65	35	0.300
4.00	65	35	0.300

## Data Availability

Not available.

## Sample Availability

Not available.
